# Enhanced electrical conductivity of TiO_2_ micro-rods through surface modification by antimony-doped tin oxide

**DOI:** 10.1039/d6ra01020b

**Published:** 2026-05-20

**Authors:** Young Seok Son, Amol Uttam Pawar, Don Keun Lee, Young Soo Kang

**Affiliations:** a Carbon Resources Conversion Research Center, Korea Institute of Energy Technology (KENTECH) 21 KENTECH-gil Naju-si Jeollanam-do 58330 Republic of Korea yskang@kentech.ac.kr

## Abstract

To explore the potential of TiO_2_ micro-rods as electrically conductive painting materials, a systematic synthesis approach was developed using an ion exchange method followed by controlled calcination. The resulting TiO_2_ micro-rods have lengths of approximately 10 µm with a diameter of nearly 250 nm. Furthermore, one-dimensional conductive TiO_2_ micro-rods coated with antimony-doped tin oxide (TiO_2_@ATO) were successfully prepared *via* a hydrothermal coprecipitation method. Cetyltrimethylammonium bromide (CTAB) was employed as a surfactant and interfacial adhesive to ensure uniform dispersion and strong surface interactions. The TiO_2_@ATO micro-rods were compression-molded under 3 tons of pressure for 3 min to form a compact disk (1.3 cm diameter), achieving a sheet resistivity of 68 Ω sq^−1^ as measured by a four-point probe technique. Morphological and compositional analyses were performed using scanning electron microscopy (SEM), transmission electron microscopy (TEM) and energy-dispersive X-ray spectroscopy (EDX), confirming the uniform coating of ATO on the TiO_2_ micro-rods. This study highlights a promising and scalable approach for producing conductive TiO_2_-based materials, suitable for use in electronic coatings and related applications.

## Abstract 2

CTAB assisted hydrothermal coprecipitation method for uniform coating of ATO on TiO_2_ micro-rods.

## Graphical abstract

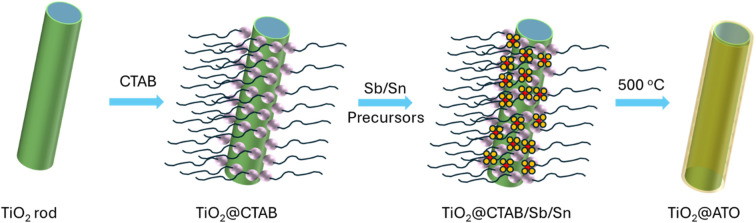

## Introduction

Titanium dioxide (TiO_2_) is a widely studied transition metal oxide known for its excellent chemical stability, non-toxicity, low cost, and high abundance. Due to these advantageous properties, TiO_2_ has found broad applications in photocatalysis, photovoltaics, gas sensing, lithium-ion batteries, and environmental remediation.^1–5^ Among the various morphologies of TiO_2_, one-dimensional structures such as rods, wires, and nanotubes have attracted particular attention for electronic and optoelectronic applications due to their high aspect ratio, efficient charge transport pathways, and anisotropic properties.^6–10^

However, a major limitation of TiO_2_, particularly in electronic or conductive applications, is its intrinsically low electrical conductivity. This limitation arises from its wide bandgap (∼3.0–3.2 eV) and poor carrier mobility, which hinder its use in conductive coatings, antistatic materials, or transparent conductors.^11^ To overcome this challenge, various strategies have been employed, such as doping with metallic or non-metallic elements, composite formation with conductive materials, and surface modification with conductive oxides.^12–15^ Among these strategies, coating TiO_2_ with antimony-doped tin oxide (ATO) has emerged as a promising approach. ATO is a highly conductive and optically transparent oxide widely used in transparent conductive films, touch screens, and solar cells.^16^ The incorporation of ATO onto TiO_2_ can enhance surface conductivity while maintaining chemical and thermal stability. Additionally, the hydrothermal co-precipitation method enables well deposition of ATO on TiO_2_ surfaces under relatively mild conditions, ensuring good adhesion and homogeneity.^17–19^ However, surfactant-assisted synthesis plays a critical role in achieving uniform and controlled coating. Various surfactants including cationic,^20^ anionic,^21^ and nonionic^22^ types have been explored for uniform coating of oxide nanostructures, the selection of an appropriate surfactant remains a key challenge. The effectiveness of a surfactant depends strongly on its molecular structure, charge characteristics, and compatibility with both host (TiO_2_) and guest (ATO) materials. A mismatch in interfacial interactions may result in poor coating uniformity, phase segregation, or deterioration of electrical properties.^23,24^ Therefore, rational selection of surfactant systems is essential to tailor interfacial chemistry and achieve desired material performance.

In this work, we present a systematic investigation of surfactant-assisted interfacial engineering in TiO_2_@ATO core–shell microrods, with particular emphasis on the role of cetyltrimethylammonium bromide (CTAB) in achieving uniform conductive coatings. Unlike conventional approaches that primarily focus on modifying optical properties, the present study demonstrates that the incorporation of ATO can significantly improve surface conductivity while preserving the intrinsic optical characteristics of TiO_2_, as evidenced by negligible changes in UV-visible and photoluminescence spectra. This decoupling of optical and electrical functionalities is highly desirable for transparent photonic and optoelectronic applications. Furthermore, a direct comparison between surfactant-assisted and surfactant-free synthesis reveals that the absence of surfactant leads to non-uniform ATO deposition, highlighting the critical role of interfacial control in determining coating homogeneity and material performance. The present work therefore provides new insights into the rational design of oxide-based hybrid structures, where surfactant-mediated assembly enables precise control over morphology, interface, and functionality. The final ATO-coated TiO_2_ micro-rods (TiO_2_@ATO) were structurally and electrically characterized using SEM, EDS, TEM, HAADF, XRD, UV, PL and four-point probe measurements.

## Experimental section

### Synthesis of TiO_2_ micro-rods

Anhydrous potassium carbonate (K_2_CO_3_, analytical grade) and anatase-phase titanium dioxide (TiO_2_, >99% purity) were used as starting materials. TiO_2_ particles were uniformly mixed with K_2_CO_3_ in deionized water at a molar ratio of *n*(TiO_2_)/*n*(K_2_CO_3_) = 3. The mixture was stirred vigorously for 3 h to ensure homogeneous dispersion, followed by drying at 200 °C to remove residual moisture. The dried powder was then subjected to calcination in a muffle furnace at 1000 °C for 8 h in air, resulting in the formation of potassium titanate (K_2_Ti_4_O_9_) micro-rods. The obtained K_2_Ti_4_O_9_ micro-rods were converted to hydrogen titanate (H_2_Ti_4_O_9_) through a proton-exchange process. Boiling it at 100 °C for 30 min and repeatedly immersing the micro-rods adjusted to pH 2 using 1.0 M HCl solution at 60 °C, followed by washing with deionized water until a neutral pH was reached. The ion-exchange step was repeated 4 times to ensure complete substitution of K^+^ with H^+^. Finally, the H_2_Ti_4_O_9_ micro-rods were calcined at 600 °C for 2 h in air to induce phase transformation, yielding TiO_2_ micro-rods. The resulting product was stored in a desiccator prior to further characterization.

### Synthesis of conductive ATO coated TiO_2_ micro-rods (TiO_2_@ATO)

To prepare ATO-coated TiO_2_ micro-rods (TiO_2_@ATO), 0.25 g of the previously synthesized TiO_2_ micro-rods was dispersed in 25 mL of deionized water under continuous stirring. CTAB was added to the suspension at a concentration of 1.0 wt%, corresponding to a weight ratio of *n*(TiO_2_)/*n*(CTAB) = 1 (0.0025 g of CTAB). The mixture was stirred at 65 °C for 1 h to ensure uniform surface modification and homogeneous dispersion of the TiO_2_ micro-rods in the solution. For the preparation of ATO precursor solution, 0.175 g of tin(iv) chloride pentahydrate (SnCl_4_·5H_2_O) was dissolved in 37.5 mL of 2.0 M hydrochloric acid (HCl), and 0.0125 g of antimony(iii) chloride (SbCl_3_) was separately dissolved in 3.75 mL of the same acid. Each solution was stirred for approximately 10 min to ensure complete dissolution before being combined to form the ATO precursor solution.

The ATO coating was carried out *via* a co-precipitation method. CTAB and TiO_2_ micro-rods were stirred together for 30 minutes and then the precursor solution and a diluted ammonia solution (1 : 3 dilution; 25% NH_4_OH in deionized water, final concentration 8.3%) were added dropwise and simultaneously to the CTAB-modified TiO_2_ micro-rods suspension under continuous stirring. During this step, the pH of the mixture was adjusted to approximately 8.5, and the co-precipitation process was carried out at temperature of 65 °C for 1 h. The resulting yellow precipitate was aged, then filtered and dried at 80 °C for 6 h. The final ATO@TiO_2_ micro-rods product was obtained by calcining the dried powder at an appropriate temperature (500 °C for 2 h) to ensure crystallization and adhesion of the ATO layer.

### Characterization

The morphologies of the TiO_2_ micro-rods and ATO-coated TiO_2_ micro-rods (TiO_2_@ATO) were examined using scanning electron microscopy (SEM, Helios G5 UX, Thermo Fisher Scientific, U.S.A) operated at an acceleration voltage of 15 kV and a working distance of 4.0 mm. Elemental composition and distribution were analyzed using energy-dispersive X-ray spectroscopy (EDS) coupled with the SEM system. The crystal structure of the TiO_2_@ATO micro-rods was investigated using X-ray powder diffraction (XRD, SmartLab 3 kV, Rigaku, Japan) and high power high resolution X-ray diffractometer (SmartLab 9 kW, Rigaku, Japan). Measurements were carried out in reflection mode using Cu-Kα radiation (*λ* = 1.5406 Å), within a 2*θ* range of 10–80°, at a scan rate of 1° min^−1^. High-angle annular dark-field scanning transmission electron microscopy (HAADF)-scanning transmission electron microscopy (STEM) images and STEM-energy dispersive X-ray spectroscopy (EDS) elemental mapping were performed at 300 kV with a double aberration-corrected STEM system (Spectra Ultra, Thermo Fisher Scientific, U.S.A) equipped with Ultra-X 6-EDS detectors. X-ray photoelectron spectroscopy (XPS, Nexsa G2, ThermoFisher, USA) with an Al Kα X-ray source (*hν* = 1486.6 eV) was employed to analyze the surface components, using a spot size of 400 µm.

To evaluate electrical properties, the resistivity of TiO_2_@ATO micro-rods was measured using a resistivity meter (ZOYI ZT-98, China). For this purpose, 0.2 g of TiO_2_@ATO micro-rods powder was compression-molded into a circular pellet (disk) with a diameter of 1.3 cm under a pressure of 3 tons for 3 min. The surface resistivity of the molded ATO@TiO_2_ micro-rods disk was further measured using a four-point probe instrument (4PX-P200) to ensure accuracy and eliminate contact resistance effects. Photoluminescence (PL) study spectra done by HORIBA FluoroMax-4 spectrofluorometer at room temperature with 350 nm excitation wavelength. The optical properties of the TiO_2_ micro-rods were characterized by UV/Vis diffuse reflectance spectroscopy (DRS) using a Cary 5000 spectrophotometer (Agilent Technologies). The optical bandgap energy was estimated *via* Tauc plot analysis of the Kubelka–Munk transformed reflectance data.

## Results and discussion

**Fig. 1:**
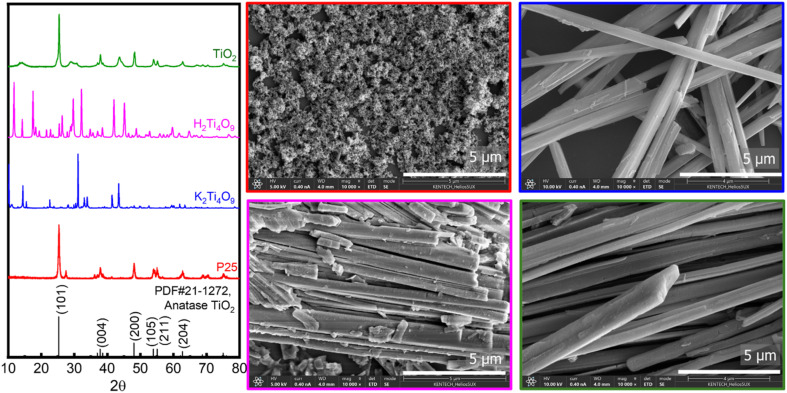
XRD and SEM data of samples P25(red), K_2_Ti_4_O_9_ (blue), H_2_Ti_4_O_9_ (pink) and TiO_2_ micro-rods (green) with standard JCPDS card of anatase TiO_2_.

The synthesis of TiO_2_ micro-rods was successfully achieved by adjusting the molar ratio of anatase TiO_2_ particles and K_2_CO_3_ precursors. This method allowed for the formation of K_2_Ti_4_O_9_ micro-rods, which were confirmed by both X-ray diffraction (XRD) and scanning electron microscopy (SEM) measurements (Fig. 1, shown in blue colour). The XRD and SEM results provided clear evidence of the successful synthesis of K_2_Ti_4_O_9_ micro-rods, confirming their characteristic crystalline structure. Following the initial synthesis, the K_2_Ti_4_O_9_ micro-rods were subjected to an ion exchange process to convert them into H_2_Ti_4_O_9_ micro-rods. The ion exchange method involves replacing potassium ions (K^+^) of the K_2_Ti_4_O_9_ structure with protons (H^+^), resulting in the formation of H_2_Ti_4_O_9_. This step is crucial for the subsequent transformations and was further confirmed through both XRD and SEM analysis, which showed the characteristic features of H_2_Ti_4_O_9_ micro-rods, indicated by the pink colour in Fig. 1. In the final step of the synthesis process, the H_2_Ti_4_O_9_ micro-rods were calcined at 600 °C in an air atmosphere. This thermal treatment was essential for converting the H_2_Ti_4_O_9_ micro-rods into anatase TiO_2_ micro-rods. Upon calcination, the H_2_Ti_4_O_9_ micro-rods structure underwent a phase transition, resulting in the formation of anatase TiO_2_, as confirmed by the XRD and SEM results as shown in Fig. 1 as green color. The transition from H_2_Ti_4_O_9_ micro-rods to anatase TiO_2_ was marked by a distinct change in the crystalline phase, which was evident in the XRD patterns. The XRD data indicated that the final anatase TiO_2_ micro-rods had a pure anatase crystal phase, without any significant impurities. This phase transition was important for the final properties of the TiO_2_ micro-rods. The SEM images of the anatase TiO_2_ micro-rods showed a well-defined rod-like morphology with an average length of more than 10 µm and a diameter of approximately 250 nm. These micro-rods exhibited a uniform and regular shape, which is ideal for many potential applications.

In conclusion, the synthesis of TiO_2_ micro-rods through the adjustment of the molar ratio of anatase TiO_2_ and K_2_CO_3_ precursors, followed by ion exchange and calcination, was successful. The final anatase TiO_2_ micro-rods exhibited the desired characteristics of length greater than 10 µm and a diameter of around 250 nm, with a pure anatase crystal phase. The XRD and SEM measurements provided strong evidence for the successful synthesis and transformation of the materials at each step. To prepare electrically conductive paint, a promising approach is to deposit antimony-doped tin oxide (ATO) on TiO_2_ micro-rods. This method involves a simple hydrothermal coprecipitation technique, where cetyltrimethylammonium bromide (CTAB) is used as a surfactant. CTAB serves as both an interfacial adhesive and a dispersing agent. In this study, we followed a previously reported method, using 1 wt% ATO and 1 wt% CTAB for the synthesis of conductive paint, specifically TiO_2_@ATO. Fig. 1a shows the comparative X-ray diffraction (XRD) spectra of pure TiO_2_ and ATO@TiO_2_. The XRD pattern of ATO@TiO_2_ clearly indicates the presence of both TiO_2_ and SnO_2_ phases, confirming the successful deposition of ATO on TiO_2_. However, there is no noticeable change in the XRD pattern due to the low concentration of Sb doping into SnO_2_, which do not have any distinct shift in the XRD peaks for the TiO_2_@ATO composite. This lack of change in the XRD pattern suggested that the Sb doping may not significantly alter the crystallographic structure of SnO_2_ in the composite.

**Fig. 2:**
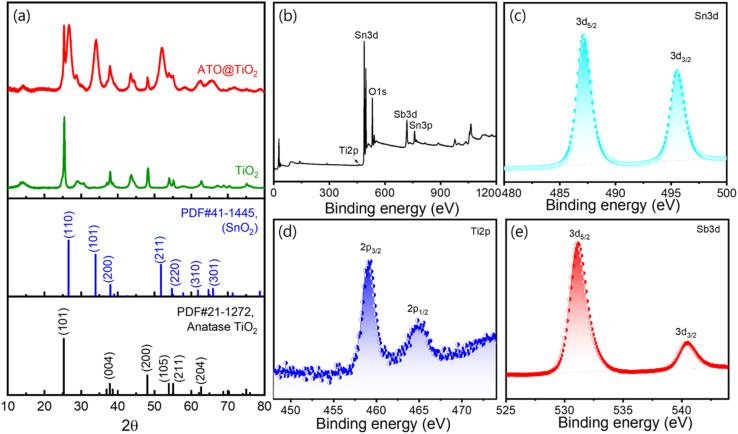
(a) XRD of TiO_2_ micro-rod (green) and Sb doped SnO deposited TiO_2_ micro-rod (TiO_2_@ATO, red). (b) XPS survey spectra, with high resolution scan of (c) Sn3d, (d) Ti2p and (e) Sb3d element of TiO_2_@ATO sample. In this case CTAB (1 wt%) and ATO (1wt%) kept constant.

To further confirm the doping of Sb into the SnO_2_ structure, X-ray photoelectron spectroscopy (XPS) measurements were carried out. The survey spectrum presented in Fig. 2b clearly shows the presence of key elements, including Sn3d, O1s, Sb3d, and Sn3p. The Ti peak is not clearly visible in the survey spectrum, which can be attributed to its low intensity of Ti element on the surface of Sb doped SnO/TiO_2_ micro-rods. This reduction in the Ti peak intensity is likely due to the coating of the TiO_2_ surface with ATO, which shields the TiO_2_ from detection. As a result, the peaks corresponding to Sb and Sn exhibit higher intensity compared to the Ti peaks, indicating the successful incorporation of Sb into the composite. To gain more insight into the chemical states and bonding of the elements in TiO_2_@ATO, high-resolution XPS scans were performed. These scans were focused on the Sn3d, Ti2p, and Sb3d peaks, which are presented in Fig. 2c–e, respectively. The high-resolution XPS spectra for Sn3d shows two distinct peaks at 487 eV (3d_5/2_) and 495 eV (3d_3/2_), corresponding to Sn in oxidation states, which is typical for SnO_2_.^25^

The Ti2p spectrum reveals the Ti^4+^ state in TiO_2_, confirming the presence of TiO_2_ in the composite with 2p_3/2_ peak at 459 eV and 2p_1/2_ at 465 eV. Similarly, the Sb3d spectrum shows clear peaks corresponding to the Sb^5+^ state from peak 3d_5/2_ at 531.1 eV and peak 3d_3/2_ at 540.5 eV, further validating the successful doping of Sb into the SnO_2_ lattice.^26^

In summary, the hydrothermal coprecipitation method successfully synthesized TiO_2_@ATO micro-rods with 1 wt% ATO and 1 wt% CTAB, using the surfactant as both an interfacial adhesive and a dispersing agent. The XRD and XPS results confirm the successful deposition of ATO on TiO_2_ micro-rods and the doping of Sb into the SnO_2_ phase. Although the XRD patterns did not show any significant changes due to the Sb doping, the XPS analysis clearly indicated the presence of Sb, Sn, and Ti in the composite, supporting the formation of TiO_2_@ATO with antimony doping.

**Fig. 3:**
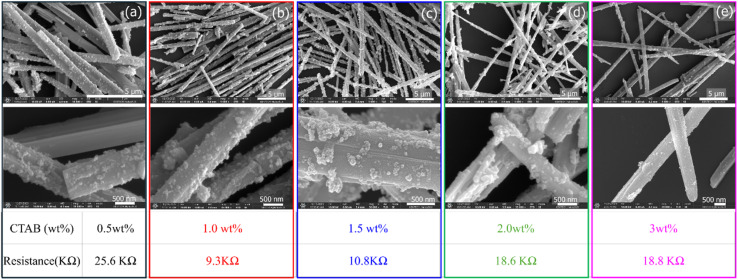
SEM images of TiO_2_@ATO micro-rods with different CTAB wt% such as (a) CTAB 0.5 wt%, (b) CTAB 1.0 wt%, (c) CTAB 1.5 wt%, (d) CTAB 2.0 wt%, (e) CTAB 3.0 wt% including their respective resistance values.

In this study, to explore the effect of CTAB surfactant concentration on the deposition of ATO on TiO_2_ micro-rods, the concentration of CTAB was systematically changed. Prior to this, the formation of TiO_2_@ATO without the use of surfactant was investigated using XRD and SEM analyses, and the results are presented in Fig. S1a–c. A detailed discussion of these results is provided in the SI. The concentrations of CTAB chosen for this experiment were 0.5 wt%, 1.0 wt%, 1.5 wt%, 2.0 wt%, and 3.0 wt%, while maintaining the ATO concentration constant at 1 wt%. The objective was to understand how different CTAB concentrations affect the uniformity and quality of the ATO deposition on TiO_2_ micro-rods and, subsequently, the electrical conductivity of the TiO_2_@ATO composite. Fig. 3 presents the SEM data, which provides a detailed view of the surface morphology of the TiO_2_@ATO composite materials at different CTAB concentrations. As the CTAB concentration increases from 0.5 wt% to 3.0 wt%, there is a noticeable increase in the deposition of ATO on the TiO_2_ micro-rods. At lower CTAB concentrations (0.5 wt%), the ATO deposition appears less uniform, and the coverage of TiO_2_ micro-rods is sparse. As the CTAB concentration increases, the deposition becomes more uniform, and the size of the ATO particles seems to become more controlled. This suggests that CTAB acts as a surfactant to disperse the ATO particles and helps to control their growth, leading to a more even coating on the TiO_2_ micro-rods.

However, determining the optimal CTAB concentration for achieving the best quality of the coated ATO layer was not straightforward from the SEM images alone. Therefore, to further evaluate the effect of CTAB concentration on the electrical properties of the composite, resistance measurements were carried out for all the samples with varying CTAB concentrations. These measurements would provide more direct insight into how the quality and uniformity of the ATO deposition influence the conductivity of the TiO_2_@ATO micro-rods. For the resistivity measurements, circular disks were prepared from samples that contained 0.2 g of TiO_2_@ATO micro-rods, which had been synthesized with different CTAB concentrations. These samples were compression-molded under a pressure of 3 tons for 3 min to form disks with a diameter of 1.3 cm. Pressures higher than 3 tons damage and deform the nanorods, making it difficult to analyze their shape accurately and reducing their natural properties. Therefore, 3 tons were selected as the best pressure to maintain the structure and stability of the film. The resistivity of the resulting disks was then measured using a resistivity meter (ZOYI ZT-98, China). The results from the resistivity measurements revealed interesting trends that helped to determine the optimal CTAB concentration.

The resistance values of the samples varied with the CTAB concentration. The maximum resistance value of 25.6 kΩ was observed for the sample prepared with 0.5 wt% CTAB. This indicates that at lower CTAB concentrations, the ATO deposition is less uniformly coated with defected surface, leading to higher resistance. The non-uniform coating may create regions with poor conductivity, which would cause an overall higher resistance. In contrast, the sample prepared with 1.0 wt% CTAB exhibited the minimum resistance value of 9.3 kΩ. This suggests that at 1.0 wt% CTAB, the ATO deposition is the most uniform coating of Sb-doped SnO_2_ on the TiO_2_ micro-rods for the optimum concentration for the highest electrical conductivity. Interestingly, as the CTAB concentration was further increased to 1.5 wt%, 2.0 wt%, and 3.0 wt%, the resistance values began to increase again. For these samples, the resistance values were 10.8 kΩ, 18.6 kΩ, and 18.8 kΩ, respectively. This indicates that while the ATO deposition for higher CTAB concentrations may have been an over-coating or aggregation of the ATO particles at concentrations above 1.0 wt%, which could lead to the formation of non-uniformity of ATO particles and reduced contact onto the surface the TiO_2_ micro-rods, resulting in higher resistance.

From these findings, it can be determined that a CTAB concentration of 1.0 wt% is the most optimum for obtaining a uniform and properly sized ATO particle coating on TiO_2_ micro-rods. This concentration provides the best balance between ATO deposition and electrical conductivity, leading to the lowest observed resistance. A concentration of 1.0 wt% CTAB allows for good dispersion of the ATO particles while avoiding issues of over-coating or aggregation that occur at higher concentrations of surfactants. The use of CTAB as a surfactant not only aids in the uniform deposition of ATO on TiO_2_ micro-rods but also plays a crucial role in optimizing the electrical properties of the final composite material. Additionally, the optical properties of pristine TiO_2_–P25 and TiO_2_@ATO composites were investigated and are presented with detailed discussion in the SI (Fig. S2).

### Mechanistic role of CTAB in ATO coating on TiO_2_

From all the obtained results role of CTAB in the formation of ATO-coated TiO_2_ micro-rods is fundamentally governed by interfacial electrostatic interactions, surface modification, and controlled heterogeneous nucleation.^27,28^ Initially, CTAB dissociates in aqueous solution into cetyltrimethylammonium (CTA^+^) cations and bromide (Br^−^) anions. The TiO_2_ micro-rods, when dispersed in water and subjected to mild heating (65 °C), present surface hydroxyl groups (Ti–OH) that partially deprotonate, generating negatively charged Ti–O^−^ sites. This enables strong electrostatic adsorption of CTA^+^ headgroups onto the TiO_2_ surface, forming a positively charged organic interface, as widely reported for CTAB-modified TiO_2_ systems.^29^ The hydrophobic alkyl chains of CTAB extend outward from the surface, producing a structured interfacial layer that acts as both a steric barrier and a soft template. Simultaneously, in the acidic precursor solution, SnCl_4_ and SbCl_3_ form stable chloro-complexes such as [SnCl_6_]^2−^ and [SbCl_4_]^−^ under high chloride concentration. These anionic complexes are electrostatically attracted to the positively charged CTAB-modified TiO_2_ surface. This interaction is consistent with established surfactant-templated coating mechanisms, where cationic surfactants mediate the deposition of inorganic shells *via* electrostatic assembly.^30,31^ Furthermore, CTAB has been shown to act as a structure-directing agent, influencing nucleation and growth behaviour in TiO_2_-based systems.^32^

Upon the controlled addition of diluted ammonia, the pH of the solution increases to approximately 8.5, triggering hydrolysis of the adsorbed metal complexes. Tin and antimony hydroxides (Sn(OH)_4_ and Sb(OH)_3_) begin to form preferentially at the CTAB-functionalized interface due to the high local concentration of precursor species. The presence of the CTAB layer suppresses homogeneous nucleation in the bulk by limiting free diffusion and stabilizing surface-bound species, leading to heterogeneous nucleation and conformal shell formation. Similar interfacial growth behavior has been observed in CTAB-derived core–shell nanostructures.^31^ During the subsequent aging process, these hydroxide intermediates undergo condensation reactions, progressively forming a mixed oxide network. Upon calcination at 500 °C, the organic CTAB layer decomposes completely, leaving behind a crystalline Sb-doped SnO_2_ (ATO) shell strongly adhered to the TiO_2_ core. The incorporation of Sb into the SnO_2_ lattice enhances electrical conductivity and promotes interfacial charge transfer, which is critical for functional applications.^30^

The concentration of CTAB plays a critical role in governing the interfacial structure and electrical properties of TiO_2_@ATO core–shell micro-rods. At concentrations below 1.0 wt%, the amount of CTAB present is insufficient to fully cover the surface of TiO_2_ rod particles. Under these suboptimal conditions, incomplete surface adsorption leads to poor dispersion, particle aggregation, and a non-uniform ATO coating on the TiO_2_ matrix. At an optimal concentration (∼1 wt%), CTAB forms a uniform monolayer or hemi micellar structure on the TiO_2_ surface through electrostatic interaction between the positively charged CTA^+^ headgroups and negatively charged Ti–O^−^ surface sites. This well-defined interfacial layer promotes the adsorption of anionic precursor complexes such as [SnCl_6_]^2−^ and [SbCl_4_]^−^, enabling controlled heterogeneous nucleation and the formation of a conformal Sb-doped SnO_2_ (ATO) shell. Such uniform coating ensures the development of a continuous conductive network, facilitating efficient charge transfer across the TiO_2_–ATO interface and resulting in low electrical resistance. However, when the CTAB concentration exceeds the optimal threshold, the interfacial chemistry and growth mechanism change significantly. Excess CTAB leads to the formation of bilayers and multilayered surfactant assemblies due to hydrophobic interactions between alkyl chains. Instead of a thin functional interfacial layer, a thicker organic film is formed on the TiO_2_ surface. Furthermore, at higher concentrations, CTAB exceeds its critical micelle concentration (CMC), leading to the formation of free micelles in the solution. These micelles compete with the TiO_2_ surface for interaction with metal precursor species, thereby reducing the effective deposition of Sn and Sb species onto the TiO_2_ surface. As a result, the ATO layer becomes discontinuous, forming isolated islands rather than a uniform conductive shell. This disrupts the percolation pathways necessary for efficient electron transport and introduces additional grain boundaries, which further increase resistive losses.^33^

### Optimization of ATO concentration

Although CTAB concentration plays an important role in regulating the coating process, it is not the sole parameter determining the uniformity, effectiveness, and reproducibility of the ATO coating. The concentration of ATO itself is equally critical for achieving a homogeneous coating with good interface quality on TiO_2_ micro-rods, which directly governs electrical stability and long-term performance. A well-controlled ATO loading not only facilitates uniform nanoparticle distribution but also improves interfacial contact between ATO and TiO_2_, thereby enhancing the overall material structure. To systematically evaluate the influence of ATO concentration, the CTAB concentration was fixed at its optimized value of 1 wt%, while the ATO concentration was varied over a range of 0.5–4.0 wt%.

**Fig. 4:**
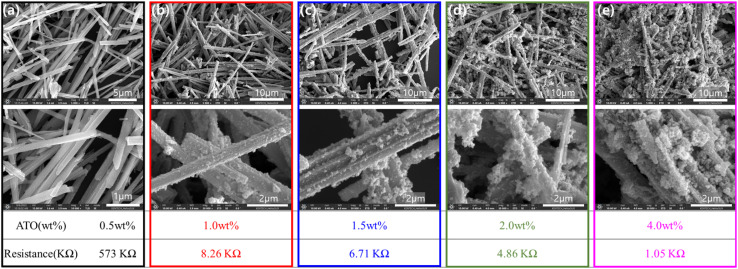
SEM images of TiO_2_@ATO micro-rods with CTAB 1.0wt% and different ATO wt% such as (a) ATO 0.5 wt%, (b) ATO 1.0 wt%, (c) ATO 1.5 wt%, (d) ATO 2.0 wt%, (e) ATO 4.0 wt% including their respective resistance values.

Fig. 4 presents SEM images of the TiO_2_ micro-rods coated with different ATO concentrations, together with their corresponding electrical resistance values. At a low ATO concentration of 0.5 wt%, the resistance is extremely high (573 kΩ), which can be attributed to incomplete and non-uniform ATO coverage. In this case, the poor coating continuity results in limited interfacial contact and discontinuous conductive pathways, leading to inferior electrical stability and poor reproducibility. As the ATO concentration increases, a pronounced decrease in resistance is observed, indicating a gradual improvement in coating uniformity and interface quality. Notably, at an ATO concentration of 1.0 wt%, the resistance decreases sharply to 8.26 kΩ. As shown in Fig. S3, this concentration marks the onset of a significant enhancement in electrical conductivity, suggesting the formation of a continuous and well-connected ATO network on the TiO_2_ surface.

Further increasing the ATO concentration to 1.5 and 2.0 wt% results in resistance values of 6.71 and 4.86 kΩ, respectively, while a minimum resistance of 1.05 kΩ is obtained at 4.0 wt%. However, the reduction in resistance beyond 1.0 wt% is relatively limited, indicating diminishing returns in electrical performance with higher ATO loadings. SEM analysis reveals that excessive ATO concentrations lead to nanoparticle agglomeration, forming dense clusters on the TiO_2_ micro-rods. Such agglomeration disrupts coating uniformity, degrades interface quality, and weakens adhesion between the ATO layer and the TiO_2_ substrate, which may adversely affect the structural integrity of the composite material.

Therefore, an ATO concentration of 1.0 wt% is identified as an optimal and cost-effective condition. At this concentration, a uniform and reproducible coating with good interface quality is achieved, providing improved electrical stability without inducing agglomeration or compromising the material structure. This balance highlights the importance of carefully optimizing ATO concentration to enhance both processing efficiency and functional performance.

To balance these trade-offs, a modified approach was considered. Instead of relying on a 1 wt% concentration of ATO, we chose to use a lower concentration specifically 0.5 wt% but apply it multiple times to the TiO_2_ micro-rods. This method aims to achieve a more uniform and stable coating without causing aggregation, thereby preserving both electrical performance and material durability.

Fig. S4 presents SEM images of TiO_2_ micro-rods coated with 0.5 wt% ATO applied once, twice, and three times. The images indicate a progressive improvement in surface coverage and coating uniformity with increasing number of ATO deposition cycles. A visual inspection confirms that the ATO layers become more continuous and homogeneous with each successive coating. This trend in coating morphology is closely correlated with a significant reduction in the measured electrical resistance of the samples. The two-probe resistance after the first ATO coating is recorded at 573 kΩ. Following the second coating, the resistance decreases sharply to 6.71 kΩ, and after the third coating, it is further reduced to 0.69 kΩ. These resistance values are systematically presented in Table S1, which summarizes the electrical measurements for all three coating stages. The results clearly indicate that increased coating cycles lead to substantial improvements in electrical conductivity. But, it should be considered in the aspect of process cost for the practical applications in industries.

**Fig. 5:**
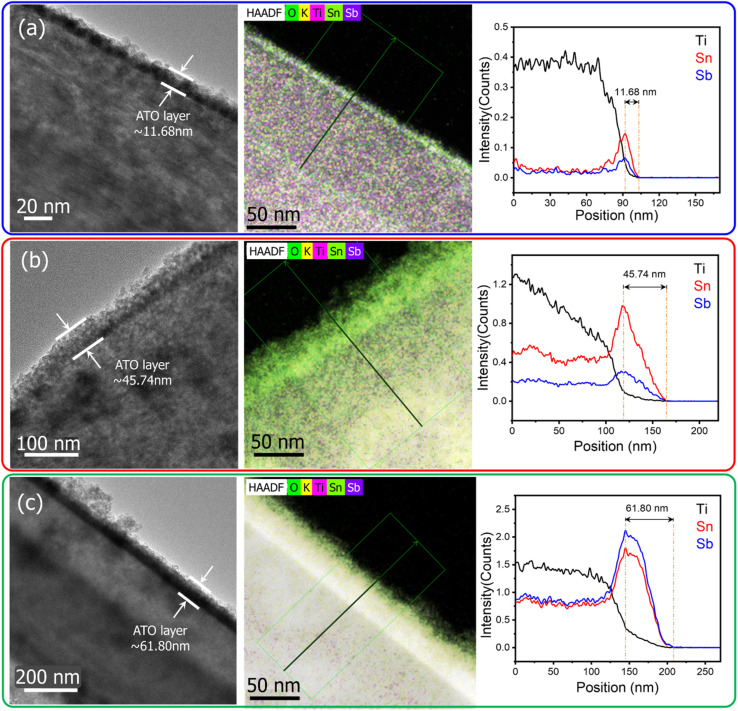
TEM with HAADF elemental line mapping of TiO_2_@ATO micro-rods with CTAB 1.0 wt% and ATO 0.5 wt% first layer coating (a), second ATO layer coating (b) and third ATO layer coating (c).

Additional structural and morphological information was obtained through transmission electron microscopy (TEM) and high-angle annular dark field (HAADF) imaging, as shown in Fig. 5. These high-resolution images reveal that all three coatings exhibit a uniform and continuous ATO layer on the TiO_2_ micro-rod surfaces. However, as the number of coating cycles increases, a corresponding increase in ATO layer thickness is observed. Specifically, the first coating results in a layer approximately 11.68 nm thick. The second coating increases this to 45.74 nm, while the third coating reaches a thickness of 61.8 nm. These results demonstrate that repeated ATO deposition cycles contribute to a thicker and more conductive coating layer. Elemental analysis *via* HAADF mapping further confirms this trend. The mapping data show a gradual increase in the elemental composition of Sn and Sb with each additional coating, validating the increased presence of ATO material across the surface. This increase in elemental concentration, combined with the greater thickness and improved uniformity, explains the enhanced conductivity observed in the electrical measurements.

To conceptually illustrate the structural evolution of the coatings, a schematic diagram is provided in Fig. S5 of the SI. This representation highlights the differences in particle size and coverage with increasing ATO layers. In the first coating, ATO nanoparticles are uniformly distributed but relatively sparse, with incomplete coverage on some surface areas due to the limited thickness. Given the inherent surface roughness of TiO_2_ micro-rods, it is likely that the initial 11.68 nm coating may not fully cover the surface, resulting in localized defects for the low conductivity and a higher overall resistance. With the second and third coatings, these empty defects are effectively filled, and the surface coverage becomes significantly more uniform. Particle size and coating density increase, forming a more continuous and conductive ATO layer. As a result, the resistance drops drastically, indicating improved electron transport through the coated structure.

The resistance of the sample was carefully measured using the four-probe technique, and the detailed results are presented in SI Table S2. This method was chosen for its superior accuracy in eliminating contact resistance, which can often interfere with the reliability of simpler methods. To ensure the reproducibility of the experiment, measurements were conducted five separate times under identical conditions. The resistance values obtained for the first, second, and third layers were approximately 6850 Ω sq^−1^, 344 Ω sq^−1^, and 68 Ω sq^−1^, respectively. These results were found to be in excellent agreement with the previously reported data, confirming the validity and consistency of the current study.

Notably, the use of the four-probe method allowed for more precise determination of sheet resistance, especially in regions with lower resistance values. Compared to the conventional two-probe technique, which can introduce significant errors due to contact and lead resistance, the four-probe setup offers a clear advantage in accuracy. This is particularly important in thin-film and layered material studies, where small variations in resistance can have a large impact on performance and interpretation.

The obtained resistance value was systematically compared with those reported in recently published TiO_2_@ATO studies, as summarized in Table 1. The results indicate that the present work exhibits a comparatively lower resistance than most of the reported values. Although a few studies demonstrate lower resistance, this discrepancy can be attributed primarily to differences in measurement techniques, such as the use of electrochemical methods to evaluate charge-transfer resistance rather than direct electrical resistance measurements. Importantly, the findings of this study highlight that electrical performance is governed not only by the intrinsic material system but also by the processing strategy and structural optimization. In contrast to previously reported approaches based on single-step deposition or high ATO loading, this work employs a multiple low-concentration ATO coating strategy. Such an approach enables precise control overcoating uniformity while effectively preserving the original TiO_2_ micro-/nanostructure. Preservation of this structural integrity is critical, as conventional high-loading or single-step methods often lead to morphological damage or pore blockage, adversely affecting charge transport. Therefore, the improved resistance observed in this study can be attributed to the synergistic effect of optimized processing conditions and controlled structural modification, demonstrating the significance of coating strategy in achieving enhanced electrical performance in TiO_2_@ATO composite systems.

**Table 1:** Comparative summary of the electrical properties of TiO_2_@ATO materials fabricated by different synthesis routes and in different material forms

No.	Materials	Synthesis method	Substrate/Form	Typical resistivity/Resistance/Electrical property	Objectives/Notes	References
1	Sb–SnO_2_ on TiO_2_	Co-precipitation	Powder composite	9–15 kΩ cm sheet resistivity	Optimized Sb/Sn & calcination; PDMS mix ∼2 MΩ	34
2	TiO_2_@Sb–SnO_2_ (core–shell)	Surface coating & calcination	Core-shell nanorods	∼52 Ω cm	Significantly reduced *vs.* pure TiO_2_ (∼10^5^ Ω cm)	35
3	TiO_2_/Sb–SnO_2_ composite	Polymeric precursor pyrolysis	Composite powder	15.42 Ω cm	Staggered heterojunction → enhanced charge transfer	36
4	TiO_2_@Sb–SnO_2_ nanocomposite	Hydrothermal	Nanocoated	∼5.97 × 10^3^ Ω cm	Ultra-fine coatings; enhanced conductivity in polymer films	37
5	TiO_2_-NTs/Sb–SnO_2_ (electrode)	Solvothermal	Ti substrate (electrode)	25 Ω (charge transfer resistance)	The charge transfer resistance reduces from 90 Ω to 25 Ω upon the introduction of TiO_2_ nanotubes arrays	38
6	TiO_2_@Sb–SnO_2_	Co-hydrolysis followed by calcination	Composites	150.3 Ω sq^−1^	Resistance decreases with increasing Sb doping concentration from 1 wt% to 5 wt%	39
7	Al_2_O_3_/ATO/TiO_2_-imbedded fabric	Synthesis by bicomponent melt spinning machine	Fabric and film	5.44 × 10^10^ Ω sq^−1^	Increasing the concentration of ATO to 70% results in lower surface electrical resistance	40
8	Sb–SnO_2_ nanonets	Coprecipitation + hydrothermal	Transparent coatings	∼0.5 kΩ per sq (on glass)	High visible transmittance (∼84.7%)	41
9	Sb:SnO_2_/TiO_2_ heterostructure	Sol–gel production followed by thin film deposition and annealing	Heterostructure film	2 × 10^3^ Ω at 300 K	The resistance of film decreases significantly with increasing operation temperature from 10^8^ Ω at 18K to 2 × 10^3^ Ω at 300 K	42
10	Sb–SnO_2_ on TiO_2_ surface (general)	Co-precipitation with particle assembly	Powder	Demonstrated reduced resistivity with uniform coating	Ti–O–Sn interface affects conductivity	43
**11**	**TiO**_**2**_ **micro-rods with ATO surface modification**	**Co-precipitation with CTAB as a surfactant**	**Composites**	**68 Ω per sq by using four probe technique**	**Promising electrical properties due to uniform coating of ATO.**	**This work**

## Conclusion

In this study, TiO_2_ micro-rods were successfully synthesized through a straightforward ion-exchange and calcination process, followed by surface modification with antimony-doped tin oxide (ATO) to enhance their electrical conductivity. The TiO_2_ micro-rods exhibited a well-defined one-dimensional morphology with a high length-to-diameter ratio, making them suitable for various applications, particularly in conductive coatings. The coating of ATO onto the TiO_2_ micro-rods was achieved using a hydrothermal co-precipitation method, facilitated by CTAB as a surfactant to ensure uniform deposition and good interfacial adhesion. The resulting TiO_2_@ATO micro-rods displayed significantly improved electrical conductivity compared to pristine TiO_2_, as confirmed by resistivity measurements and four-point probe testing. XRD, SEM, TEM and HAADF analyses revealed that the ATO coating was successfully adhered to the surface of the TiO_2_ micro-rods without altering their structural integrity.

These findings suggest that TiO_2_@ATO micro-rods possess promising electrical properties, making them viable candidates for use in applications such as conductive paints, antistatic coatings, and electronic devices. The straightforward and scalable synthesis method, coupled with the enhanced conductivity of the ATO-coated TiO_2_ micro-rods, opens up new possibilities for industrial-scale applications of these materials in various electronic and energy-related fields. Future work could focus on further optimizing the coating process and exploring the long-term stability and performance of these materials in real-world applications.

## Conflicts of interest

There are no conflicts to declare.

## Supplementary Material

RA-016-D6RA01020B-s001

## Data Availability

All experimental data are included in the article and its supplementary information (SI). Supplementary information is available. See DOI: https://doi.org/10.1039/d6ra01020b.
