# Supplementary material for: Enhanced electrical conductivity of TiO2 micro-rods through surface modification by antimony-doped tin oxide
Source: RSC Adv. 2026 May 20;16(28):25881–91. doi: 10.1039/d6ra01020b (PMC13191743; doi:10.1039/d6ra01020b)
Supplement: RA-016-D6RA01020B-s001 [file RA-016-D6RA01020B-s001.pdf]

Supplementary Information (SI) for RSC Advances.
This journal is © The Royal Society of Chemistry 2026

Supporting Information

Enhanced Electrical Conductivity of TiO_2 Micro-Rods Through Surface Modification by Antimony-doped Tin Oxide

Young Seok Son, Amol Uttam Pawar, Don Keun Lee, and Young Soo Kang

Carbon Resources Conversion Research Center, Korea Institute of Energy Technology (KENTECH), Naju, Jeollanamdo, 58217, Republic of Korea.

AUTHOR INFORMATION

Corresponding Author

E-mail: yskang@kentech.ac.kr

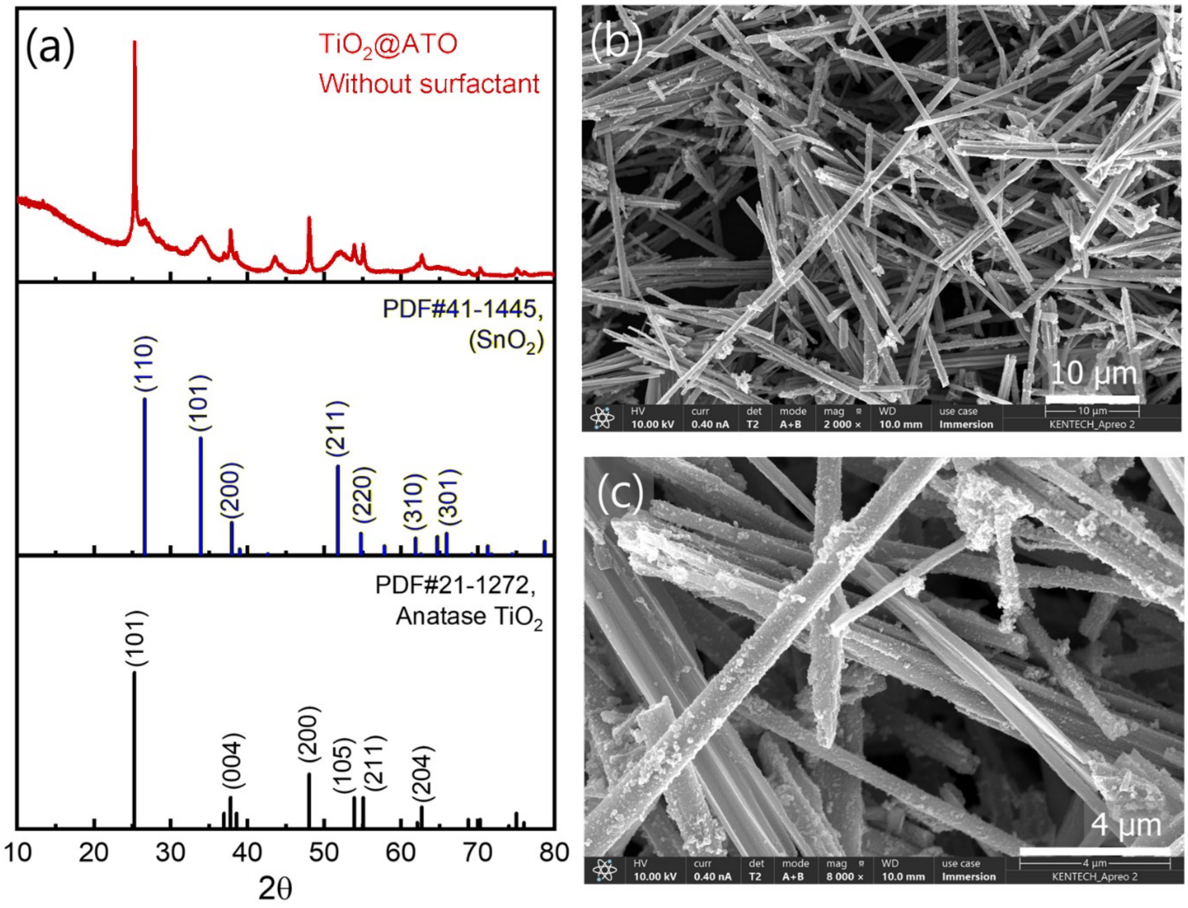

Figure S1. (a) XRD spectra of prepared TiO_2@ATO micro-rods without surfactant with JCPDS data of SnO_2 and Anatase TiO_2. SEM images of prepared TiO_2@ATO without surfactant with lower (b) and higher (c) magnification.

Figure S1 presents the XRD patterns of the synthesized TiO_2@ATO microrods prepared in the absence of any surfactant. As observed, the characteristic diffraction peaks corresponding to ATO exhibit very low intensity compared to those of TiO_2, indicating that the formation or deposition of the ATO phase is limited under these conditions. This weak signal suggests that ATO is either poorly crystallized or unevenly distributed on the TiO_2 surface, resulting in insufficient surface coverage. The absence of a surfactant likely leads to uncontrolled nucleation and growth of ATO particles, causing aggregation in localized regions rather than uniform coating across the microrods.

This interpretation is further supported by the SEM images shown in Figure S1b and S1c. The micrographs clearly reveal that the surface of the TiO_2 microrods lacks a consistent and homogeneous ATO layer. Instead, irregular and scattered deposits can be observed, confirming that ATO nanoparticles are not uniformly anchored onto the TiO_2 framework. Such non-uniform coating may adversely affect interfacial contact and limit the enhancement of electrical or functional properties expected from the TiO_2@ATO composite system.

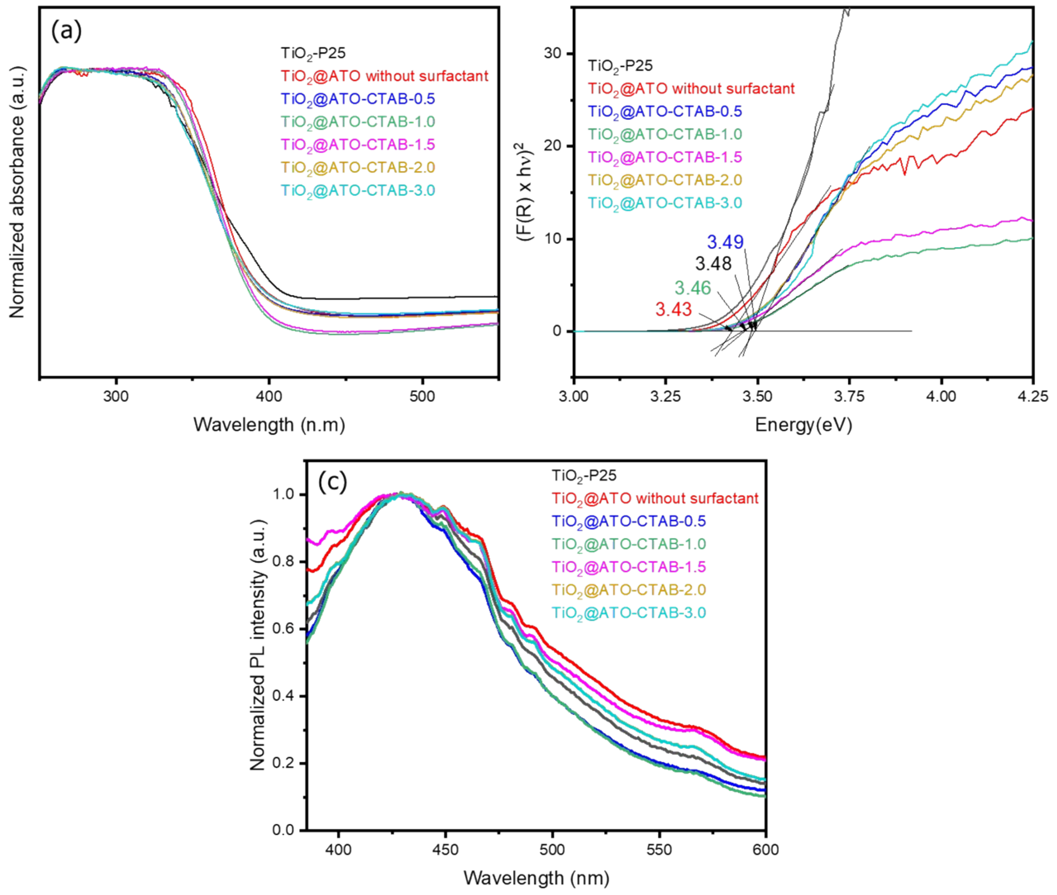

Figure S2. (a) UV-absorbance, (b) Tauc plot and (c) Photoluminescence spectra of pristine TiO_2, TiO_2@ATO without surfactant and TiO_2@ATO with the variation of CTAB surfactant.

The UV–vis absorption spectra (Figure S2a) of pristine TiO_2-P25 and TiO_2@ATO composites with varying CTAB concentrations (0–3.0 wt%) were analyzed to evaluate their optical properties. All samples exhibit strong absorption in the UV region (≈250–400 nm), corresponding to the intrinsic band-to-band transitions of TiO_2 from the valence band to the conduction band. [1,2] The presence of a well-defined absorption edge in all cases confirms that the fundamental semiconductor characteristics of TiO_2 are retained after ATO incorporation with CTAB assisted synthesis.

The optical band gaps were estimated using Tauc plots (Figure S2b). Pristine TiO_2 shows a band gap of approximately 3.48 eV. Upon incorporation of ATO without the use of surfactant, the band gap slightly decreases to 3.43 eV, which can be attributed to morphological variations, such as the formation of relatively larger TiO_2 nanorods compared to P25 nanoparticles. In the presence of CTAB, the band gap values remain nearly unchanged, with a value of 3.46 eV observed at 1.0 wt% CTAB and only negligible variation at higher concentrations. A slight increase to 3.49 eV is observed at 0.5 wt% CTAB. These results indicate that neither ATO incorporation nor CTAB-assisted synthesis significantly alters the intrinsic electronic structure of TiO_2.

The absence of any notable red shift or visible-light absorption suggests that ATO does not act as a sensitizer but rather serves as a conductive phase. Additionally, CTAB primarily facilitates improved dispersion and uniform coating of ATO on the TiO_2 surface, particularly at an optimal concentration of 1.0 wt%. Therefore, the observed modifications are predominantly interfacial rather than electronic in nature. Overall, the UV–vis analysis demonstrates that the enhanced functional performance of TiO_2@ATO composites is unlikely to originate from changes in optical absorption, but rather from improved charge separation and transport enabled by the conductive ATO network and optimized interfacial contact.

Figure S2c presents the normalized photoluminescence (PL) emission spectra of pristine TiO_2-P25 and TiO_2@ATO composites, recorded under an excitation wavelength of 350 nm. All samples exhibit a dominant emission band centered at approximately 430 nm, which is characteristic of near-band-edge recombination in TiO_2, along with contributions from shallow trap states such as oxygen vacancies and surface defects.[3] The PL intensity reflects the rate of radiative recombination of photogenerated electron–hole pairs. Compared to pristine TiO_2, the TiO_2@ATO composites exhibit reduced PL intensity, indicating suppressed charge carrier recombination due to improved interfacial charge transfer. Notably, the sample prepared with 1.0 wt% CTAB shows the lowest PL intensity among all compositions, suggesting the most efficient separation of photogenerated charge carriers. This improvement can be attributed to the role of CTAB as a structure-directing agent, which enhances the ATO uniform and intimate interfacial contact with TiO_2, thereby facilitating electron transfer.

However, further increasing the CTAB concentration beyond 1.0 wt% (1.5–3.0 wt%) results in a gradual increase in PL intensity, indicating a decline in charge separation efficiency. This behavior suggests that excessive surfactant may disrupt the optimal interfacial interaction between TiO_2 and ATO, due to forming isolated islands rather than a uniform layer which hinder effective electron transport.

Overall, the observed trend demonstrates that an optimal CTAB concentration is critical for achieving efficient interfacial engineering in TiO_2@ATO composites. Moderate surfactant content (1.0 wt%) enhances charge separation, whereas excess CTAB adversely affects electron transfer, leading to increased recombination. These results highlight the importance of controlled surfactant-assisted synthesis in optimizing the photophysical properties of TiO_2-based heterostructures.

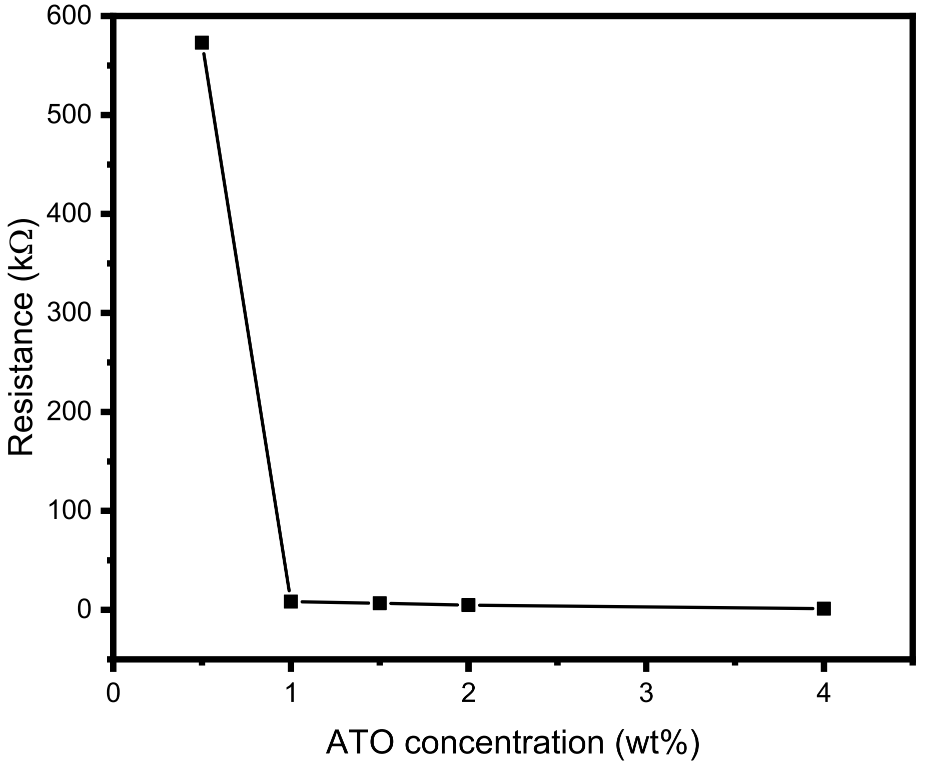

Figure S3. Resistance values of TiO_2 micro-rods prepared at different ATO concertations.

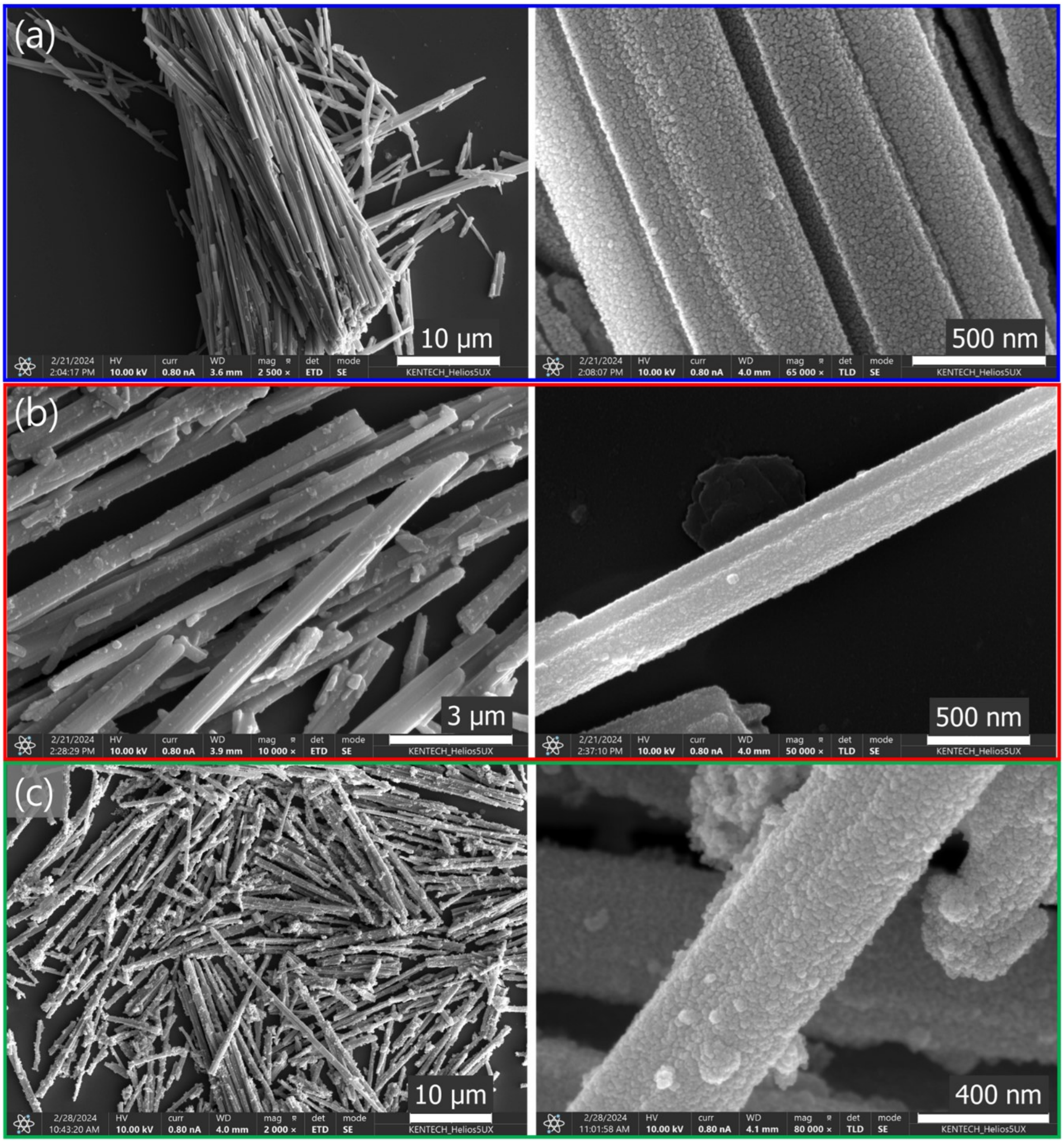

Figure S4. SEM data of lower and higher magnification for 0.5 wt% ATO coating on the surface of TiO_2 micro-rods for the (a) first time, (b) second time and (c) third time.

Table S1. Resistance value of TiO_2 micro-rods with 0.5 wt% ATO coating at different times, with CTAB concentration held constant at 1wt%.

**ATO solution (wt%)**	ATO x 0.5wt%	ATO x 0.5wt%	ATO x 0.5wt%
**Coating**	1st time	2nd time	3rd time
**Resistance(KΩ)**	573	6.71	0.696

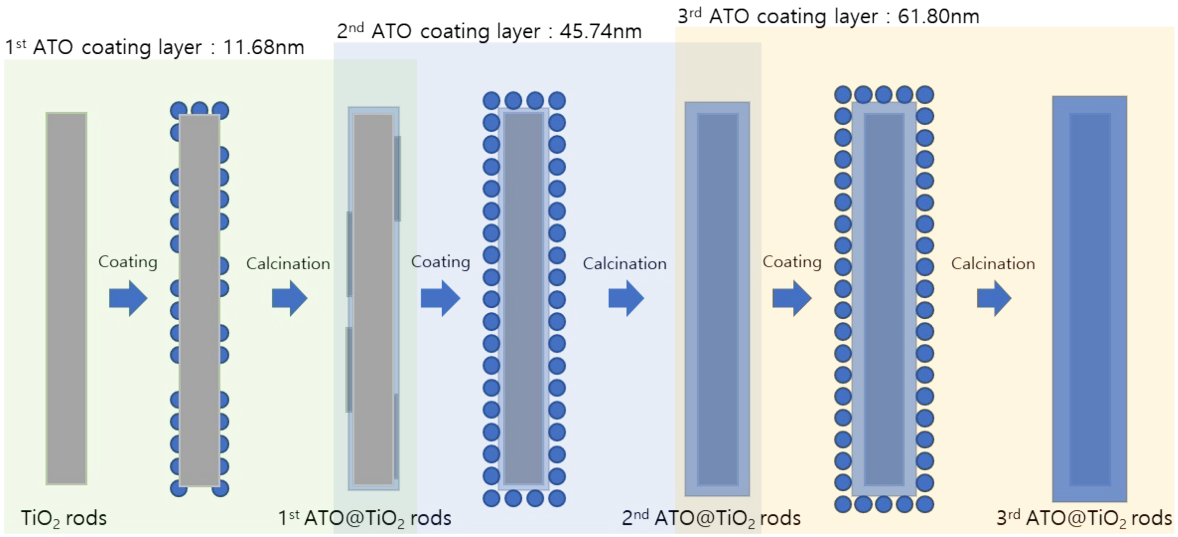

Figure S5. Schematic representation of 0.5 wt% ATO coating on the surface of TiO_2 micro-rods in three different times when CTAB kept constant as 0.5wt%.

Table S2. The careful resistance of ATO coated TiO_2 micro-rods measured by sophisticated four probe techniques for five different samples prepared with same experimental condition

CTAB(wt%)	ATO(wt%)	Sheet Resistance (Ω/sq)					
		1	2	3	4	5	Average
1.0	0.5	6474	6814	6770	7026	7167	6850
	1.0(0.5/0.5)	344	344	342	346	344	344
	1.5(0.5/0.5/0.5)	69	71	67	67	67	68.2

and check the results reproducibility.

References

1. A. Fujishima, X. Zhang, D. A. Tryk, *Surface Science Reports,* 2008, **63**, 515-582.
2. U. Diebold, *Surface Science Reports,* 2003, **48**, 53-229.
3. A. Saha, A. Moya, A. Kahnt, D. Iglesias, S. Marchesan, R. Wannemacher, M. Prato, J. J. Vilatela, D. M. Guldi, *Nanoscale*, 2017, **9**, 7911-7921.
